# Construction Notes

**Published:** 1893-11-25

**Authors:** 


### CONSTRUCTION NOTES.

### Haydock Cottage Hospital.

The new wing to this hospital has been completed and furnished, bringing the total amount of accommodation in the institution up to sixteen beds. The committee had hoped to open the new addition to the institution free of debt, but they have not been able to effect this, £242 only being required to make up the deficit on the building accounts. Lord Newton has granted the use (at a merely nominal rate) of a piece of land behind the hospital, which, when fenced in and laid out, will be a great benefit to convalescent patients.

### New Convalescent Home at Hexham.

This new convalescent home of the Cathedral Nurse and Loan Society, Newcastle, was recently opened; it is pleasantly situated at the south end of Hexham. It is built of Choppington bricks, and has been erected by Messrs. James Herdman and Son, of Hexham, from designs by Mr. A. Crawford-Hick, of Newcastle, the contract amount being £2,470, exclusive of furnishing. There is accommodation for 21 patients, in addition to that for the matron and servants. The home is for the reception of women and children only, that at Shotley Bridge being set apart for men. All bed and living rooms face south. The sum of £800 is still wanted to clear the building of debt.

### Extension of Leeds Hospital for Women and Children.

Owing to the great increase in the number of out-patients attending this hospital during the past two years the waiting-room was found to be too small for their accommodation. The committee have added, therefore, a wing to the east of the main building, at a cost of £400. The new structure is 62 feet long, 21 feet wide, and 18 feet high. In connection with it there is also a matron's office and lobby. The rooms are well ventilated, and heated by the Hygienic Condensing Stove. The former waiting-room has been converted into a waiting-room, store-room, and lavatory. The extension was designed by Mr. Connon (Messrs. Chorley and Connon, of Park Row, Leeds), under whose superintendence the work has been carried out.

